# The First Whole-Genome Characterization of a Kenyan DS-1-like G3P[8] Rotavirus Strain: Evidence for an Intragenogroup Reassortment Event in Africa

**DOI:** 10.3390/v18090930

**Published:** 2026-08-24

**Authors:** Yuki Akari, Aoko J. Ogutha, Maurine M. Mutua, Mary Wachira, Carlene Sang, Saori Fukuda, Ryoko Shiraishi, James Nyangao, Samoel A. Khamadi, Shingo Inoue, Satoshi Kaneko, Ernest A. Wandera, Satoshi Komoto

**Affiliations:** 1 Division of One Health, Research Center for GLOBAL and LOCAL Infectious Diseases (RCGLID), Oita University, Yufu 879-5593, Oita, Japan; m24d9001@oita-u.ac.jp (Y.A.); m25d9109@oita-u.ac.jp (M.M.M.); shiraishir@oita-u.ac.jp (R.S.); 2 Department of Medical Microbiology, Jomo Kenyatta University of Agriculture and Technology, Nairobi P.O. Box 62000-00200, Kenya; pauljohnogutha@gmail.com; 3 Kenya Research Station, Nagasaki University Institute of Tropical Medicine (NUITM)-KEMRI, Nairobi P.O. Box 19993-00202, Kenya; mnwachira3@gmail.com (M.W.); pampanga@nagasaki-u.ac.jp (S.I.); skaneko@nagasaki-u.ac.jp (S.K.); ewandera@kemri.go.ke (E.A.W.); 4 Department of Environmental Health and Disease Control, Jomo Kenyatta University of Agriculture and Technology, Nairobi P.O. Box 62000-00200, Kenya; 5 Centre for Virus Research, Kenya Medical Research Institute (KEMRI), Nairobi P.O. Box 54840-00200, Kenya; csang@kemri.go.ke (C.S.); jnyangao2000@yahoo.com (J.N.); skhamadi@gmail.com (S.A.K.); 6 Department of Virology, Fujita Health University School of Medicine, Toyoake 470-1192, Aichi, Japan; saorif@fujita-hu.ac.jp; 7 Center for Infectious Disease Research, Research Promotion Headquarters, Fujita Health University, Toyoake 470-1192, Aichi, Japan; 8 Innovation and Technology Transfer Division, KEMRI, Nairobi P.O. Box 54840-00200, Kenya

**Keywords:** *Rotavirus alphagastroenteritidis*, whole-genome analysis, DS-1-like G3P[8] strains, intragenogroup reassortment, genomic diversity, Kenya

## Abstract

Unusual DS-1-like G3P[8] rotavirus strains have emerged and spread rapidly across several countries. In Africa, however, reports of these strains and available whole-genome data remain limited, and their evolutionary relationships across the continent are not yet fully understood. In this study, we sequenced and characterized the complete genome of a DS-1-like G3P[8] strain (RVA/Human-wt/KEN/KCH1748/2020/G3P[8]) detected in a child with acute gastroenteritis in Kenya. Strain KCH1748 possessed an unusual genotype constellation: G3-P[8]-I2-R2-C2-M2-A2-N2-T2-E2-H2. Phylogenetic analysis revealed that 10 of the 11 genomic segments of strain KCH1748 were closely related to those of other East African DS-1-like G3P[8] strains from Kenya and Tanzania within the globally circulating DS-1-like G3P[8] lineage, suggesting that it may be derived from this globally emerging lineage. In contrast, the VP1 gene of strain KCH1748 was closely related to those of Ghanaian G9P[4] strains, sharing a common branch with Beninese DS-1-like G3P[8] and G2P[4] strains, suggesting a VP1 intragenogroup reassortment event involving African RVA strains. This study provides the first comprehensive whole-genome evolutionary characterization of a DS-1-like G3P[8] strain identified in Kenya. Our findings contribute to understanding the evolutionary dynamics and genomic diversification of emerging DS-1-like G3P[8] strains in Africa.

## 1. Introduction

*Rotavirus alphagastroenteritidis* (RVA), a member of the *Rotavirus* genus in the *Sedoreoviridae* family, is a leading cause of severe acute gastroenteritis (AGE) in children worldwide. RVA-associated disease is estimated to cause 128,500–215,000 annual deaths among children aged <5 years, primarily in sub-Saharan Africa [1,2]. Despite this high burden of RVA disease, data on genotype diversity and whole-genome sequences of RVA strains remain limited in Africa, particularly in sub-Saharan African countries [3,4].

The RVA genome consists of 11 segments of double-stranded RNA (dsRNA). RVA strains are traditionally classified based on the two outer capsid proteins, VP7 and VP4, which define the G and P genotypes, respectively [5]. To date, 42 G and 58 P genotypes have been recognized by the Rotavirus Classification Working Group (RCWG) (https://rega.kuleuven.be/cev/viralmetagenomics/virus-classification/rcwg, accessed on 1 August 2026). Human RVA strains typically belong to G1P[8], G2P[4], G3P[8], G4P[8], G9P[8], and G12P[8] genotypes, although their prevalence varies geographically and temporally [6]. A whole-genome classification system based on all 11 genomic segments was established using the nomenclature Gx-P[x]-Ix-Rx-Cx-Mx-Ax-Nx-Tx-Ex-Hx [7]. The majority of human RVAs are assigned to two genotype constellations: Wa-like (G1/3/4/9/12-P[8]-I1-R1-C1-M1-A1-N1-T1-E1-H1) and DS-1-like (G2-P[4]-I2-R2-C2-M2-A2-N2-T2-E2-H2) [8,9]. In polyacrylamide gel electrophoresis (PAGE) analysis, these genogroups are characterized by distinct dsRNA migration patterns corresponding to long (Wa-like) and short (DS-1-like) electropherotypes [10,11].

In Kenya, RVA-associated disease remains a major cause of pediatric morbidity and mortality despite the introduction of the monovalent RVA vaccine Rotarix^®^ (G1P[8]; GlaxoSmithKline Biologicals, Rixensart, Belgium) into the national immunization program in July 2014 [12,13,14]. Following the introduction of Rotarix^®^, surveillance studies in Kenya documented an increase in the proportion of G3P[8] among circulating RVA strains [15,16]. At the multi-site level, the prevalence of G3P[8], which shares the P[8] specificity with Rotarix^®^, increased from 1.3% (8/614) in the pre-vaccine period to 16.1% (42/261) in the early post-vaccine period [15]. In central Kenya, a long-term surveillance study further showed that G1P[8], which shares both G1 and P[8] specificities with Rotarix®, initially remained prevalent after vaccine introduction, whereas G3P[8] became dominant during the late post-vaccine period (2019–2020), representing 61.1% (140/229) of strains, which can be compared with 13.1% (30/229) for G1P[8] [16]. However, the genomic characteristics and evolutionary origin(s) of the emerging G3P[8] strains in Kenya remain poorly understood.

In recent years, unusual DS-1-like G3P[8] RVA strains have emerged globally. These strains are intergenogroup reassortants characterized by an equine-like G3 gene, which has recently been suggested to be related to bat-like RVA sequences [17], on a DS-1-like genomic backbone. These strains were first identified in Thailand and Australia in 2013 [18,19]. Subsequently, these strains disseminated rapidly across Asia, Europe, and the Americas, often becoming predominant among circulating RVA strains in several countries [11,20,21,22,23,24,25,26,27,28,29].

In Africa, however, DS-1-like G3P[8] strains have been identified only in Kenya, Tanzania, and Benin [30,31,32]. These strains were first identified in Kenya in 2018, when G3P[8] was the predominant genotype, accounting for 67.2% (37/48) of the strains [32]. Based on phylogenetic analysis of the VP7 and VP4 genes, two Kenyan G3P[8] strains shared close relationships with globally circulating DS-1-like G3P[8] strains. Although the complete genome sequence of one of these strains, KLF0918, was subsequently deposited in public databases, its genomic evolutionary relationships have not yet been described. Subsequently, two DS-1-like G3P[8] strains, Mbeya-MHO32 and Mbeya-MHO37, were identified among eight RVA-positive samples in neighboring Tanzania [31]. These Tanzanian strains shared close relationships with strain KLF0918 and globally circulating DS-1-like G3P[8] strains, based on analysis of the 10 available genomic segments, suggesting the introduction and regional spread of DS-1-like G3P[8] strains in East Africa. More recently, three DS-1-like G3P[8] strains, 20P093, 22011, and 22013, were identified in Benin, West Africa, in 2020 and 2022 [30]. Phylogenetic analyses identified segment-specific genetic diversity among these strains, particularly in the NSP2 genomic segment. Collectively, these reports indicate that DS-1-like G3P[8] strains related to globally circulating lineages have emerged in Africa. However, their evolutionary relationships and genomic diversification require further elucidation.

Here, we performed whole-genome characterization of KCH1748, a Kenyan DS-1-like G3P[8] strain identified during post-vaccine surveillance in 2020 [16]. We also sequenced two co-circulating human RVA strains, a Wa-like G3P[8] strain (KCH1884) and a DS-1-like G2P[4] strain (KCH194), to provide regional genomic references for evaluating the evolutionary relationships of the G3P[8] genotype and the DS-1-like genomic backbone.

## 2. Materials and Methods

### 2.1. Sample Selection and Virus Strains

The RVA strains analyzed in this study were obtained from stool specimens collected from children hospitalized with AGE during post-vaccine surveillance conducted at Kiambu County Teaching and Referral Hospital (KCTRH) in Central Kenya between 2019 and 2020, during which G3P[8] was the predominant genotype, accounting for 61.1% (140/229) of RVA strains [16]. To identify potential DS-1-like G3P[8] strains, 80 G3P[8]-positive specimens with sufficient residual volume were screened by PAGE analysis. Based on this screening, KCH1748 was the only strain with a short RNA electropherotype consistent with a DS-1-like genomic backbone and was therefore selected for whole-genome sequencing and characterization. Two co-circulating RVA strains, the Wa-like G3P[8] strain KCH1884 and the DS-1-like G2P[4] strain KCH194, were included for comparative genomic analysis.

Strains KCH1748, KCH1884, and KCH194 were identified from three diarrheic children aged 15 months (male; March 2020), 8 months (male; September 2020), and 18 months (female; February 2019), respectively. Clinically, all three individuals presented with watery and mucoid diarrhea and had previously completed the two-dose Rotarix^®^ vaccination schedule. The study protocol and sample collection were approved by the Scientific and Ethical Review Unit (SERU) of the Kenya Medical Research Institute (KEMRI) (SCC No. 1323). Additionally, written informed consent was obtained from the parents or legal guardians of all the participating children.

### 2.2. Genomic dsRNA Extraction and Full-Length RT-PCR Amplification

Genomic dsRNA was extracted from 10% stool suspensions using a QIAamp Viral RNA Mini Kit (QIAGEN, Hilden, Germany). For strain KCH1748, all 11 RVA genomic segments were amplified using segment-specific full-length RT-PCR before next-generation sequencing. Briefly, cDNA was synthesized from the extracted dsRNA, after which each genomic segment was amplified separately to full length using segment-specific primers, as described previously [33,34]. The resulting PCR products were purified using the Wizard^®^ SV Gel and PCR Clean-Up System (Promega, Madison, WI, USA) and subsequently subjected to DNA library preparation. In contrast, extracted viral dsRNA from strains KCH1884 and KCH194 was directly subjected to library preparation.

### 2.3. Library Preparation and Next-Generation Sequencing

For strain KCH1748, purified full-length PCR products corresponding to the 11 RVA genomic segments were pooled and subjected to DNA library preparation using the NEBNext Ultra II FS DNA Library Prep Kit (New England Biolabs, Ipswich, MA, USA) and purified with NEBNext Sample Purification Beads (New England Biolabs, USA). For the comparator strains KCH1884 and KCH194, cDNA libraries were constructed directly from extracted viral dsRNA using the NEBNext Ultra II RNA Library Prep Kit (New England Biolabs, USA) and purified with NEBNext Sample Purification Beads, as previously described [19,35]. Following the assessment of the quality and quantity of the purified cDNA library, 151-cycle paired-end sequencing was conducted using a MiSeq sequencer (Illumina, USA) with the MiSeq Reagent Kit v2 (Illumina, San Diego, CA, USA). The obtained FASTQ files were analyzed using CLC Genomics Workbench version 8.0.1 (CLC Bio, Aarhus, Denmark). Contigs were generated from sequence reads after adapter trimming using de novo assembly. The assembled contigs were used as query sequences in the Basic Local Alignment Search Tool (BLAST, version 2.17.0+) (https://blast.ncbi.nlm.nih.gov/Blast.cgi?PROGRAM=blastn&PAGE_TYPE=BlastSearch&LINK_LOC=blasthome, accessed on 28 May 2026) on the National Center for Biotechnology Information (NCBI) platform to identify contigs corresponding to the full-length or near-full-length sequences of each genomic segment of the study strains. To enhance the quality of the contigs, the sequence reads of individual genomic segments were mapped back to the assembled contigs. RVA nucleotide sequences were translated into amino acid sequences using GENETYX v11 (GENETYX, Tokyo, Japan).

### 2.4. Determination of RVA Genotypes and Phylogenetic Analysis

The genotypes of all 11 genomic segments were assigned using the Rotavirus A Genotyping Tool v0.1 provided by the Rijksinstituut voor Volksgezondheid en Milieu (RIVM) (https://www.rivm.nl/mpf/typingtool/rotavirusa/, accessed on 28 May 2026) and BLAST. The percentage of nucleotide sequence identity between the study strains was determined using the FASTA program with GENETYX software [36]. Multiple sequence alignments of each genomic segment were performed using the MUSCLE algorithm [37], which was implemented in MEGA software package version 12.1 [38]. Phylogenetic trees based on maximum likelihood estimation were constructed for all 11 genomic segments. For each genomic segment, the reference dataset included representative globally circulating DS-1-like G3P[8] strains from previous whole-genome studies, the most closely related sequences identified by BLAST searches, and African RVA strains selected to assess regional phylogenetic relationships. The best substitution model for each segment was selected based on the corrected Akaike information criterion (AICc) implemented in MEGA12.1. The models used in this study were Tamura 3-parameter (T92) + gamma distributed (G) + invariable sites (I) (VP7 and VP4), General Time Reversible (GTR) + G + I (VP1 and VP3), Tamura-Nei (TN93) + G + I (VP6 and VP2), T92 + I (NSP1 and NSP5), and Hasegawa-Kishino-Yano (HKY) + G + I (NSP2, NSP3, and NSP4). The reliability of the branching order was evaluated using 1000 bootstrap replicates. The mVISTA online platform was used to visualize nucleotide sequence similarities between the concatenated genome sequence of strain KCH1748 and those of representative RVA strains: the Thai prototype DS-1-like G3P[8] strain SKT-281, the Kenyan DS-1-like G3P[8] strain KLF0918, the Tanzanian DS-1-like G3P[8] strain Mbeya-MHO32, the Beninese DS-1-like G3P[8] strain 20P093, and the Ghanaian G9P[4] strain 039M. The deduced amino acid sequences of all 11 RVA proteins were aligned using CLUSTAL W provided in GENETYX version 11.

### 2.5. Nucleotide Sequences

The nucleotide sequence data obtained in this study have been deposited in the DDBJ/ENA/GenBank databases. The accession numbers for the nucleotide sequences of the VP1–VP4, VP6, VP7, and NSP1–NSP5 genomic segments of the study strains are LC940160–LC940170 for KCH1748, LC940171–LC940181 for KCH1884, and LC940182–LC940192 for KCH194, respectively.

## 3. Results

### 3.1. Whole-Genome Sequencing and Genotype Constellations of the Study Strains

PAGE-based screening of the 80 available G3P[8] strains identified KCH1748 as the only strain with a short RNA electropherotype, consistent with a DS-1-like genomic backbone. Illumina MiSeq sequencing of pooled full-length RT-PCR amplicons from strain KCH1748 generated approximately 1.5 × 10^6^ reads with an average length of 141.1 bp, enabling determination of the full-length nucleotide sequences of all 11 genomic segments. Direct sequencing of extracted viral dsRNA from the two comparator strains generated approximately 9.0 × 10^5^ reads with an average length of 149.9 bp for strain KCH1884 and 1.9 × 10^6^ reads with an average length of 146.5 bp for strain KCH194. The lengths of the nucleotide sequences and encoded proteins for each genomic segment and the sequence read coverage data are summarized in Supplementary Table S1.

The 11 genomic segments of strain KCH1748 were assigned the genotype constellation G3-P[8]-I2-R2-C2-M2-A2-N2-T2-E2-H2 (Table 1). Strain KCH1748 was confirmed as an unusual DS-1-like G3P[8] strain, based on its G3P[8] genotype and short RNA electropherotype. Strain KCH1748 was designated as RVA/Human-wt/KEN/KCH1748/2020/G3P[8] according to the guidelines for RVA nomenclature uniformity proposed by the RCWG [39]. In contrast, the 11 genomic segments of the two co-circulating strains, KCH1884 and KCH194, possessed typical genotype constellations. Strain KCH1884 possessed a Wa-like genotype constellation, G3-P[8]-I1-R1-C1-M1-A1-N1-T1-E1-H1, and was designated as RVA/Human-wt/KEN/KCH1884/2020/G3P[8]. Strain KCH194 possessed a DS-1-like G2P[4] constellation, G2-P[4]-I2-R2-C2-M2-A2-N2-T2-E2-H2, and was designated as RVA/Human-wt/KEN/KCH194/2019/G2P[4].

### 3.2. Phylogenetic Analysis and Whole-Genome Similarity Comparisons

**Figure 1:**
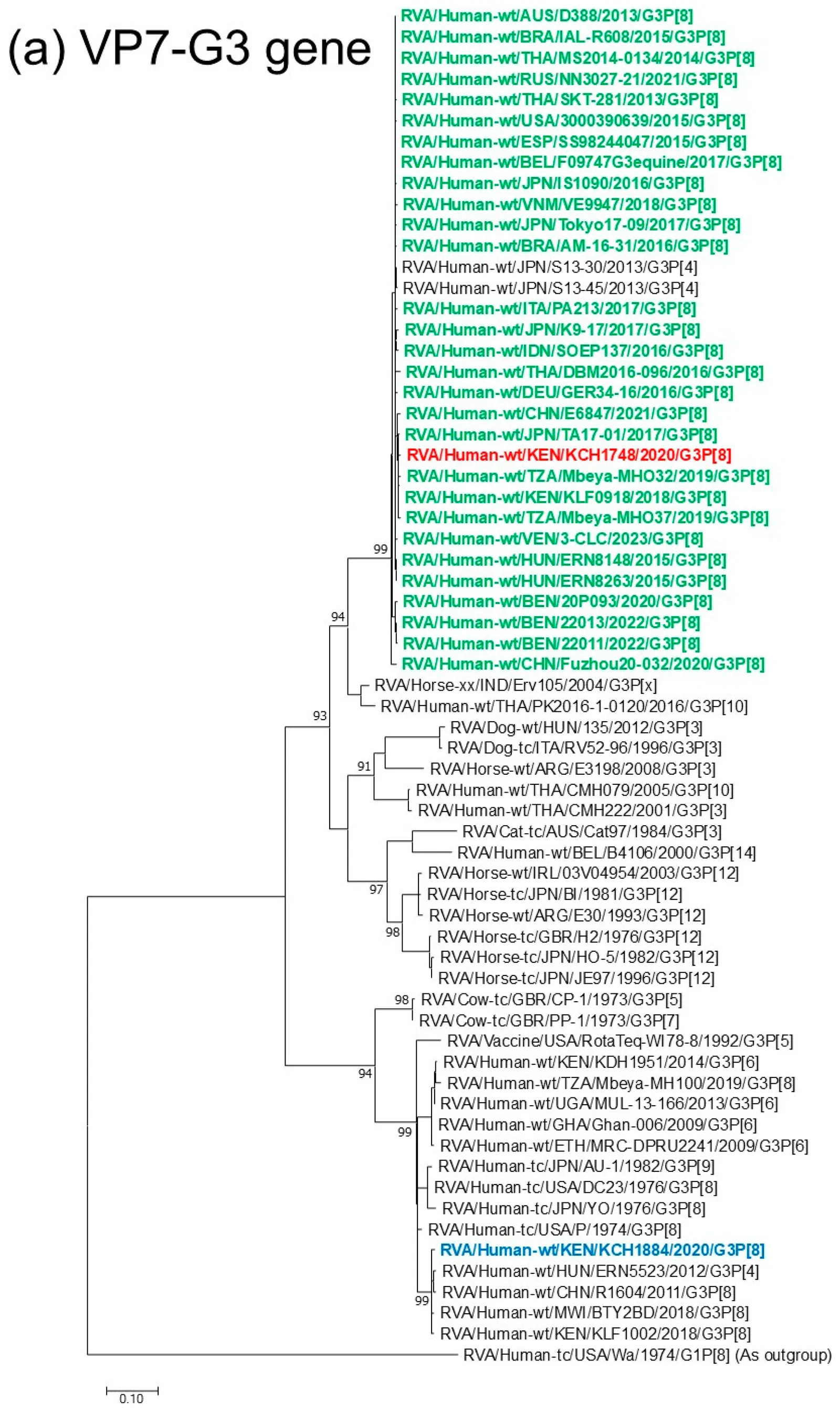

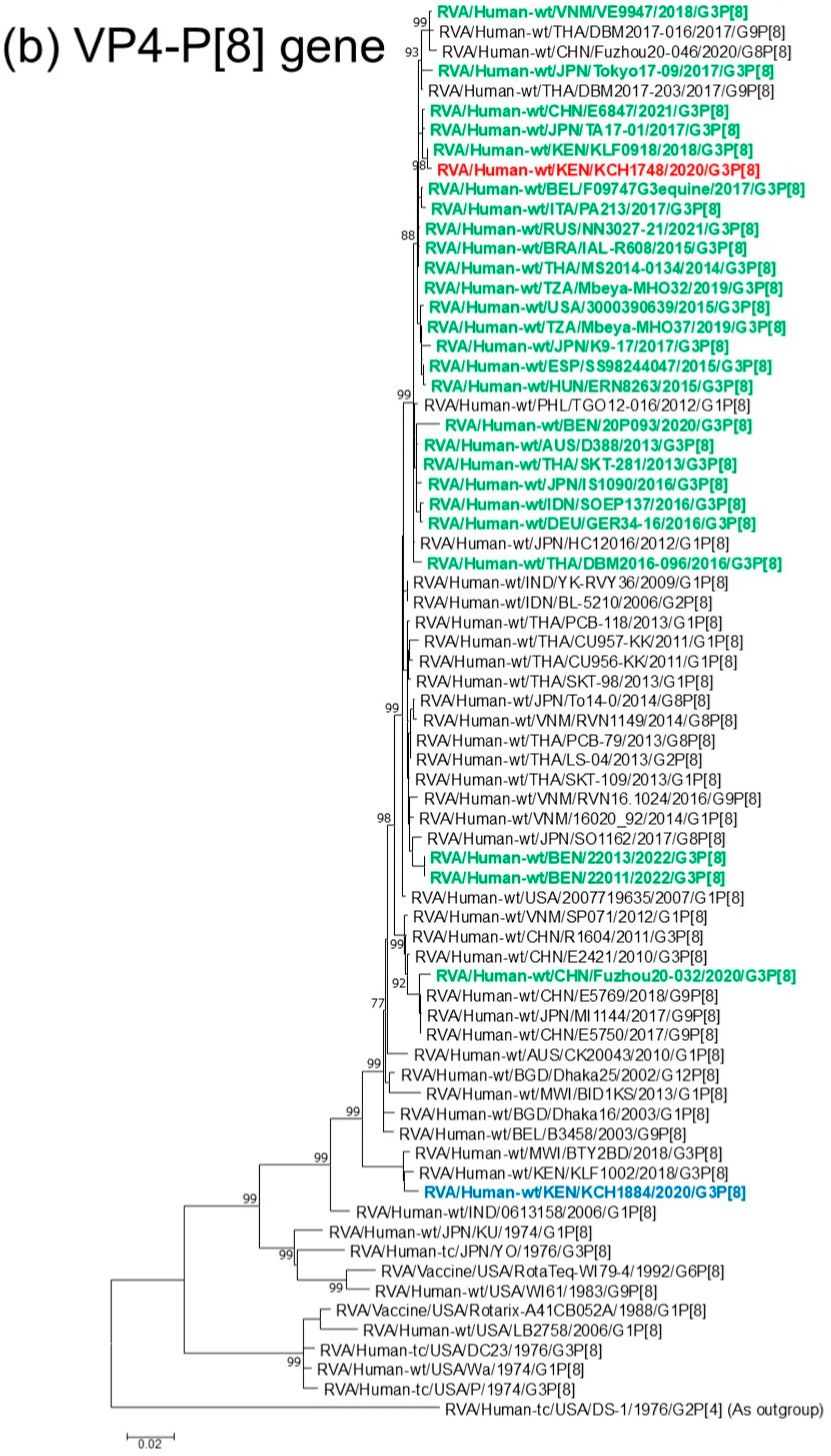

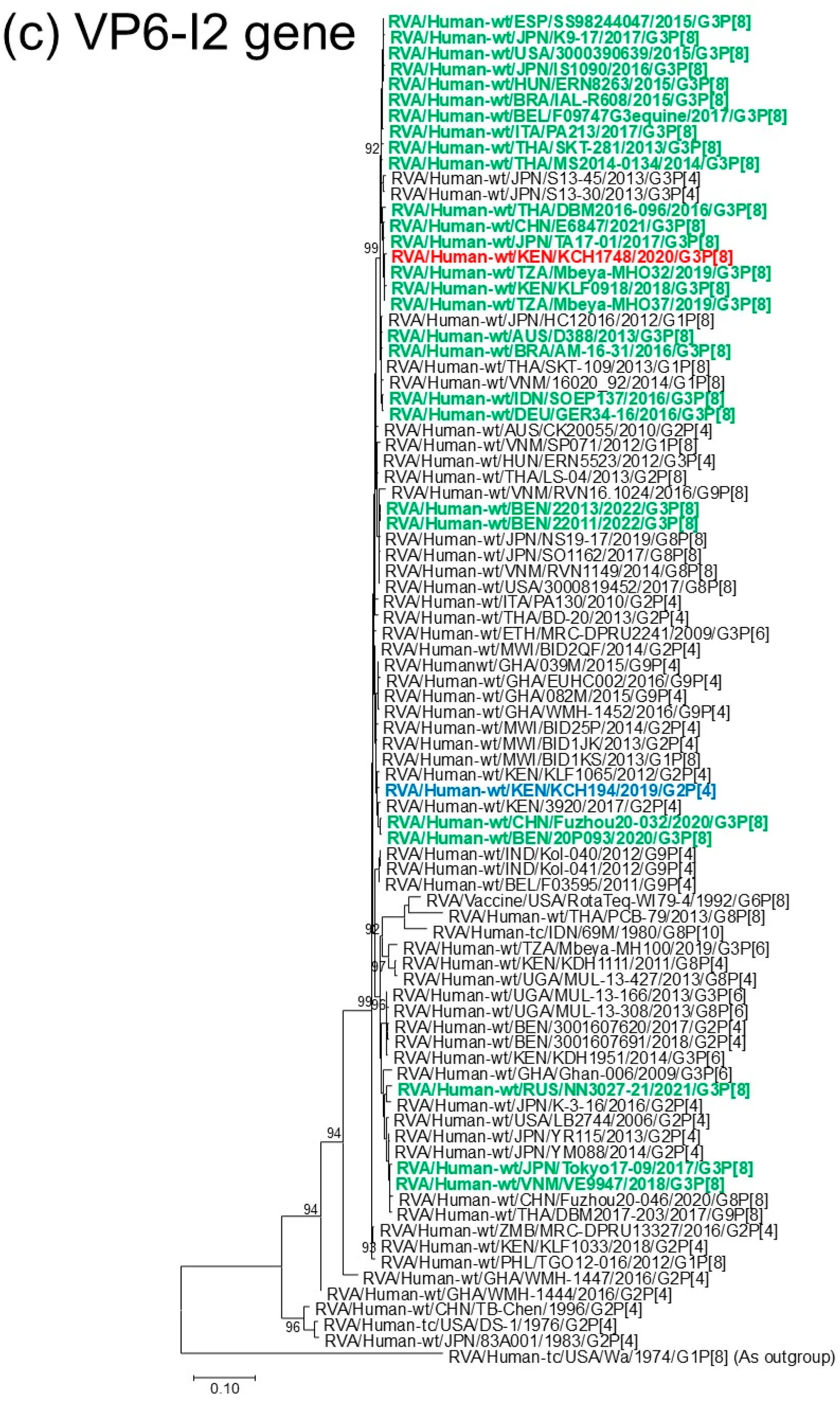

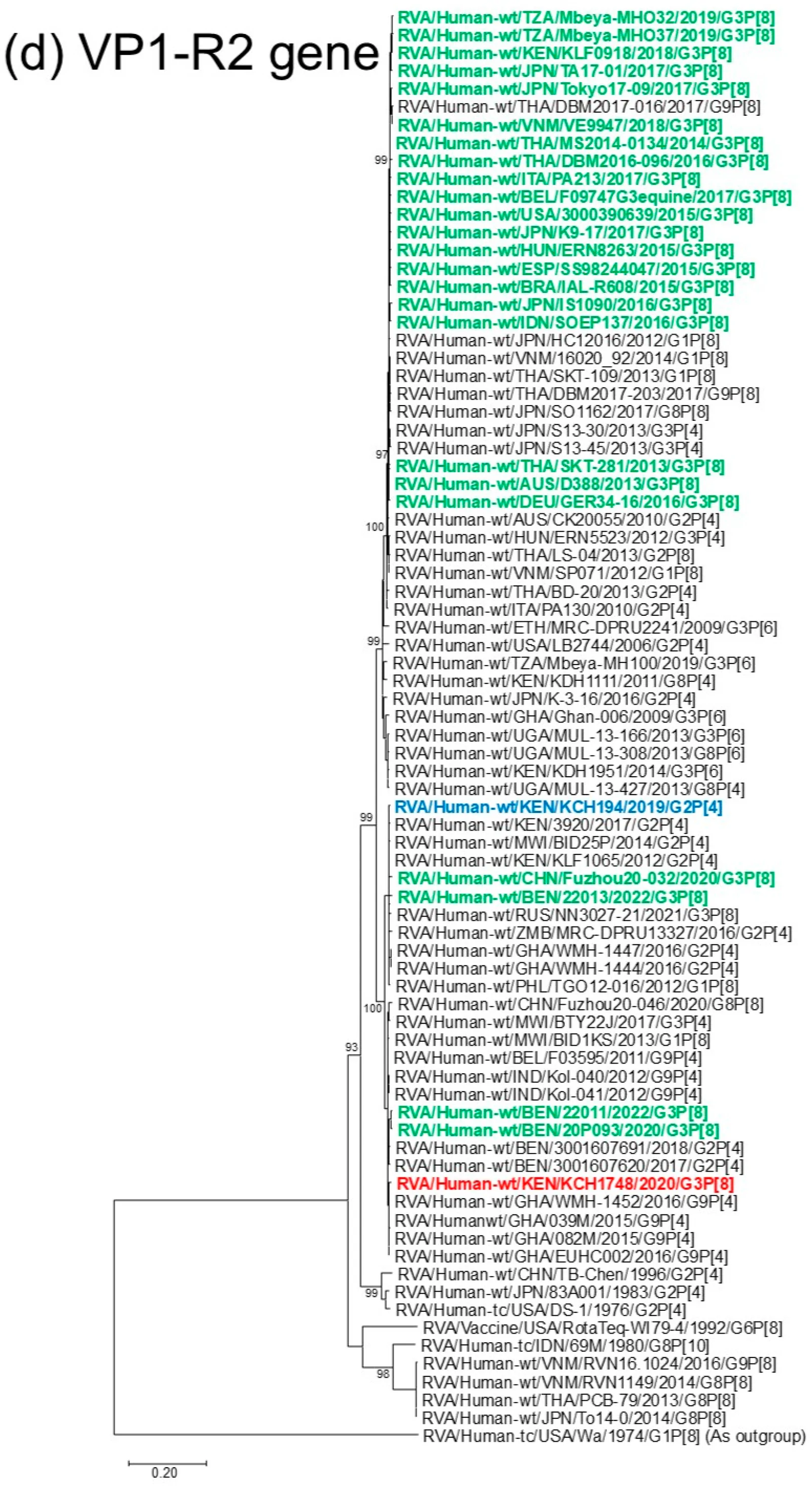

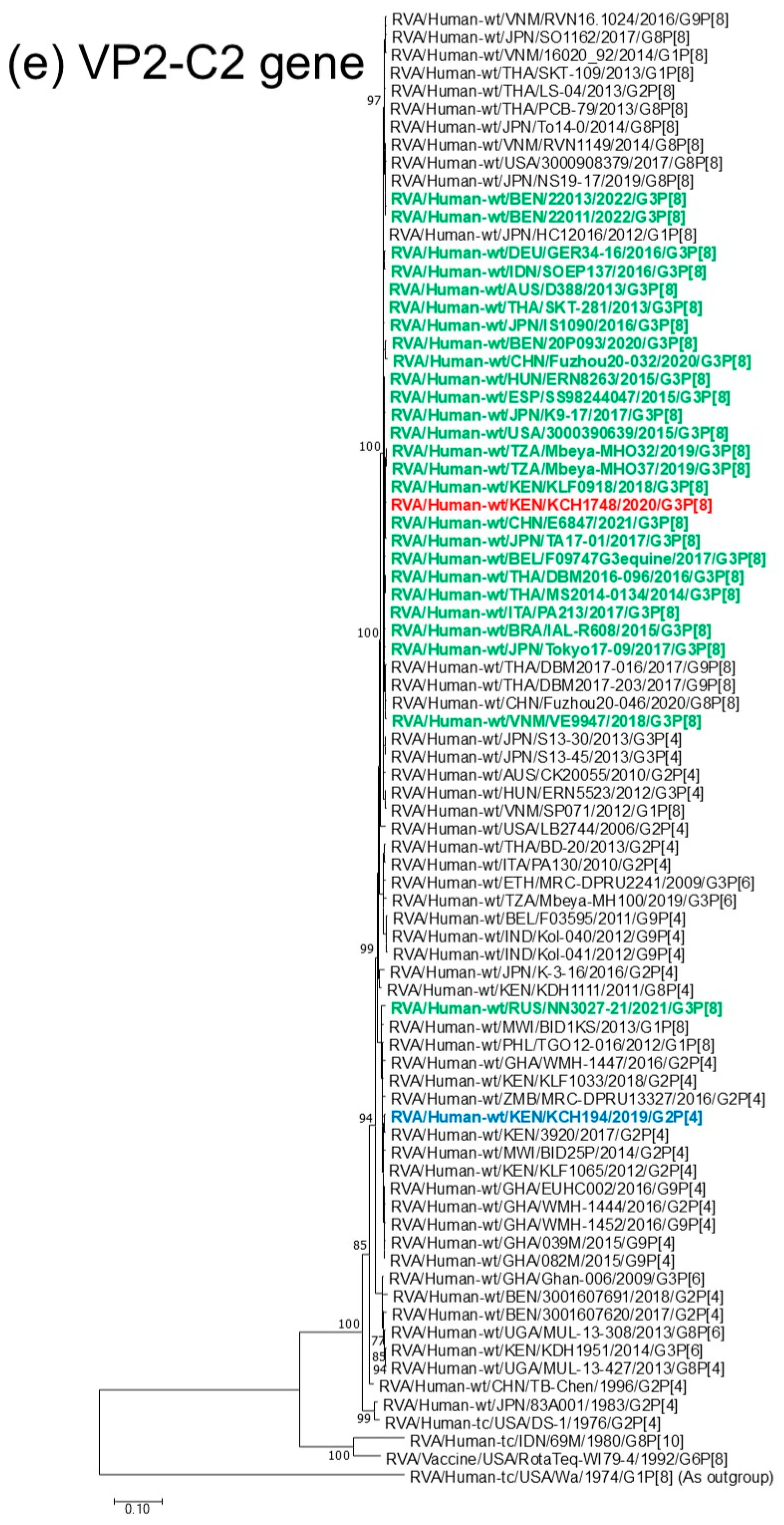

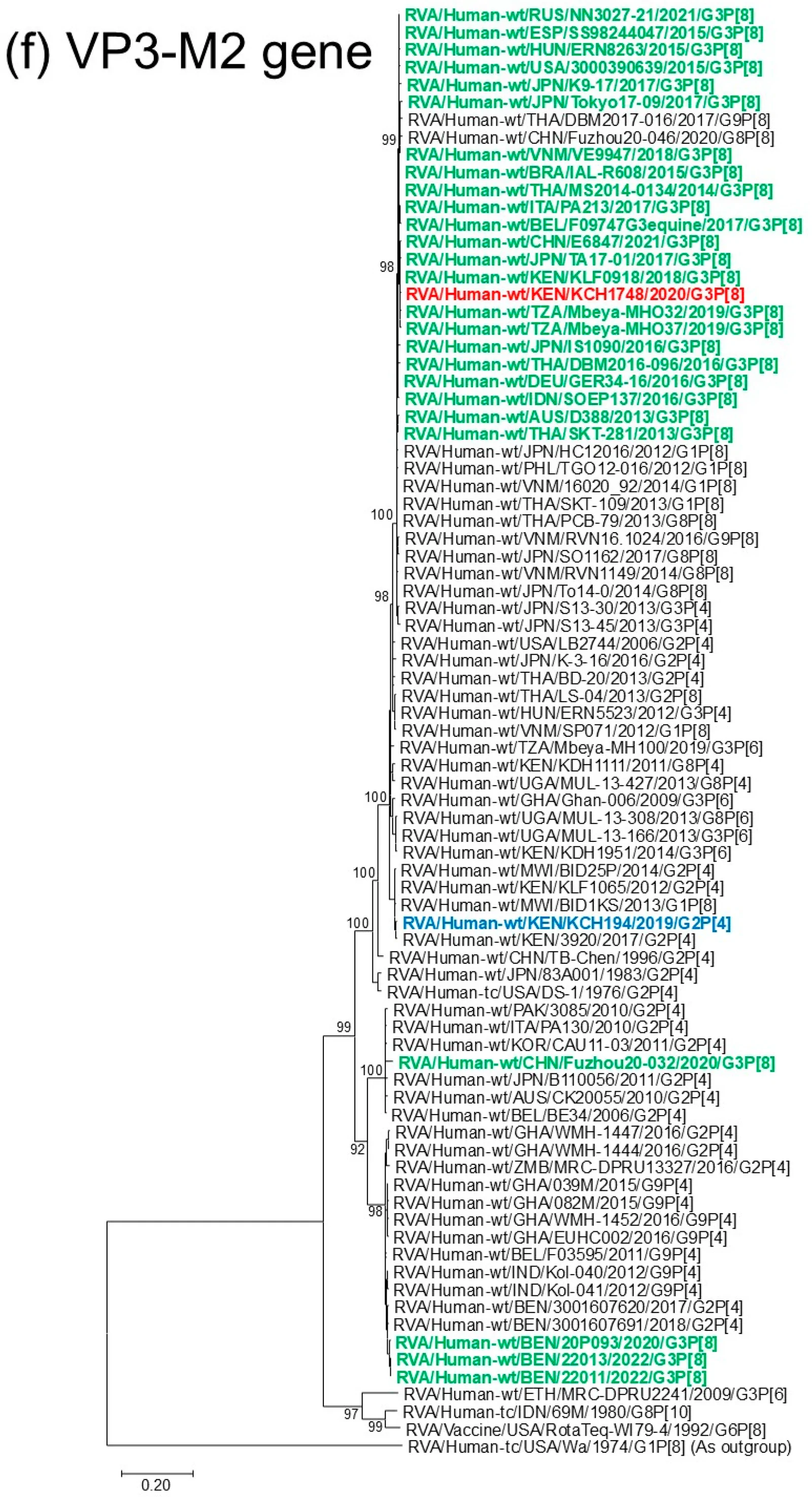

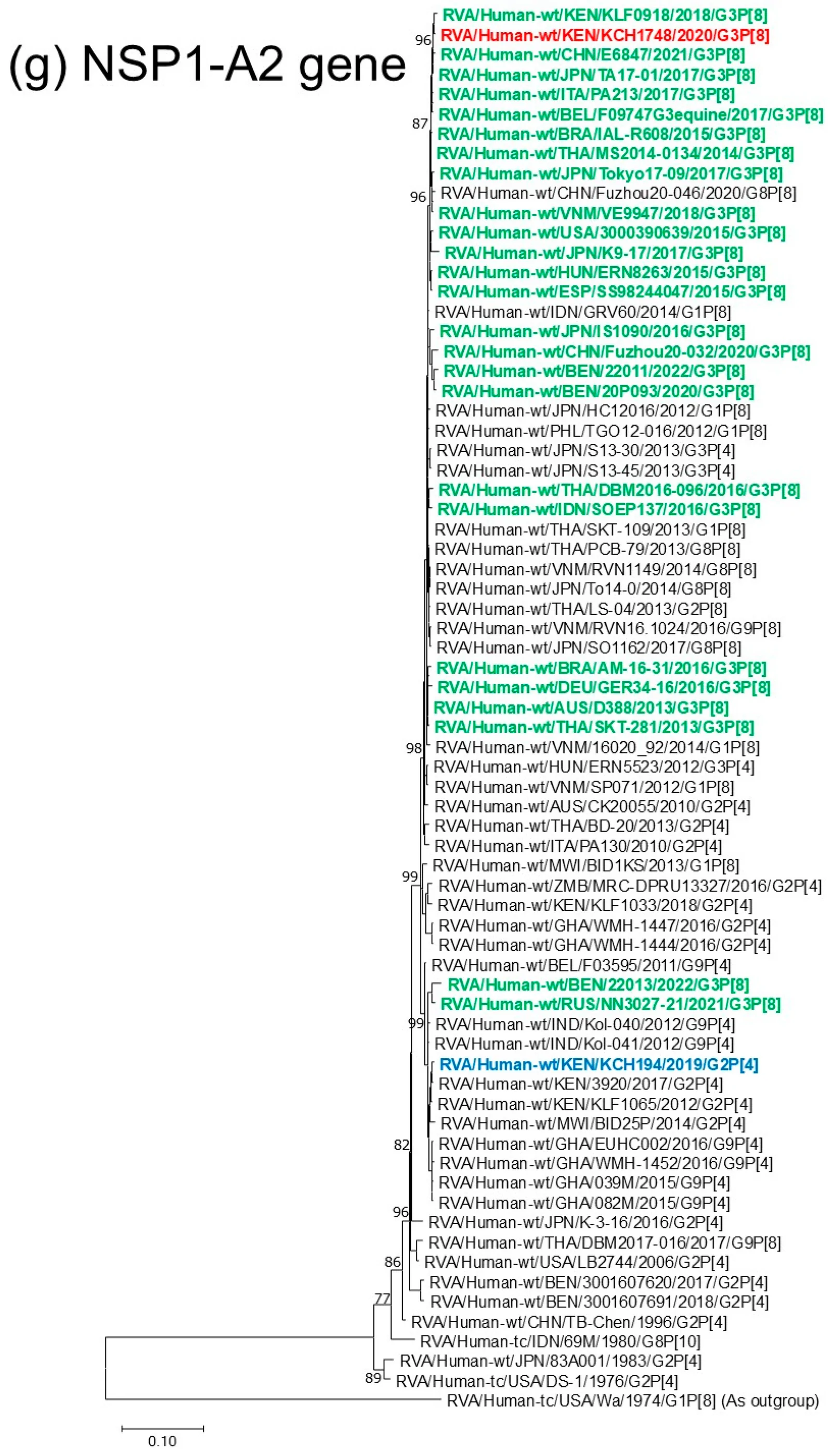

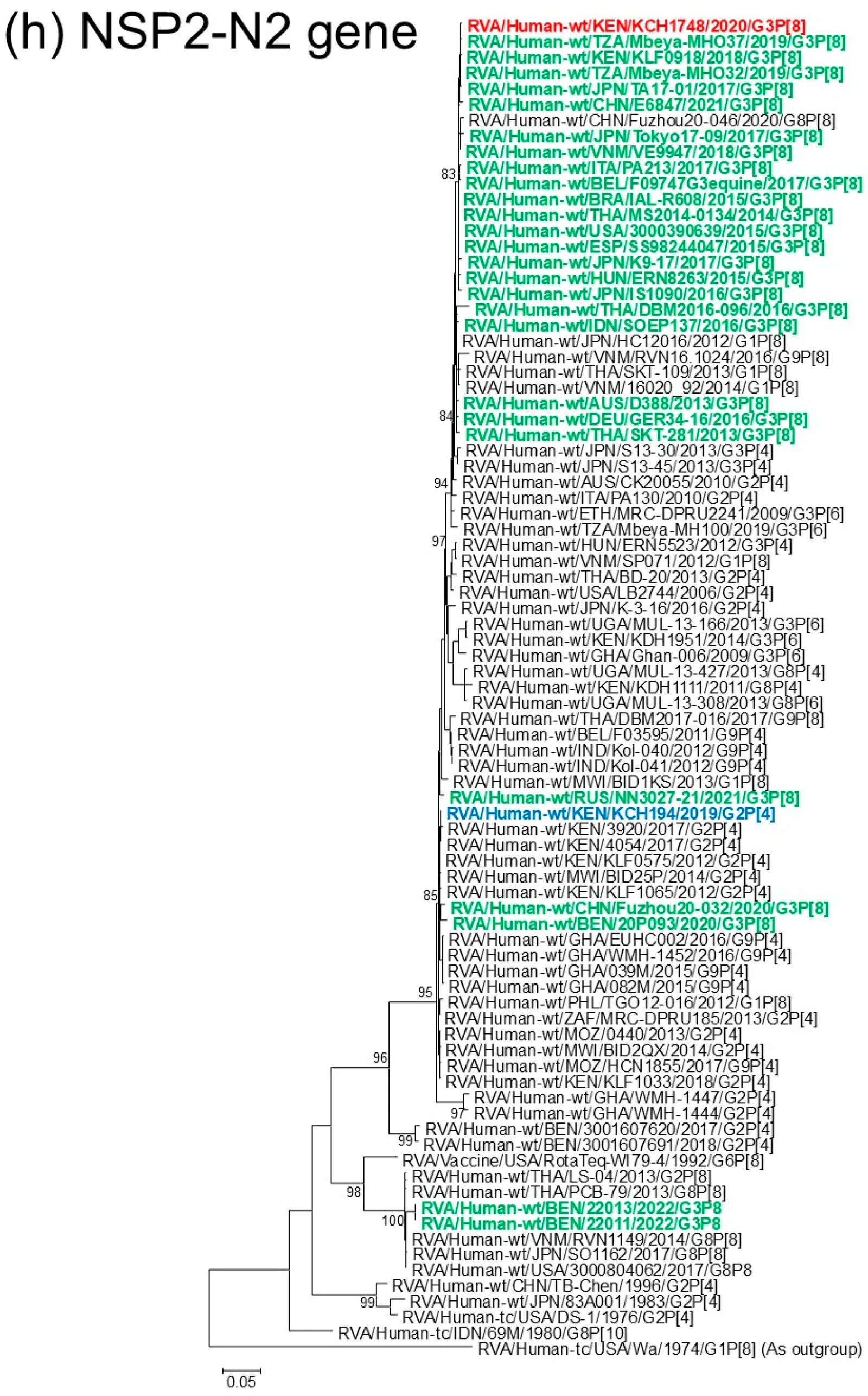

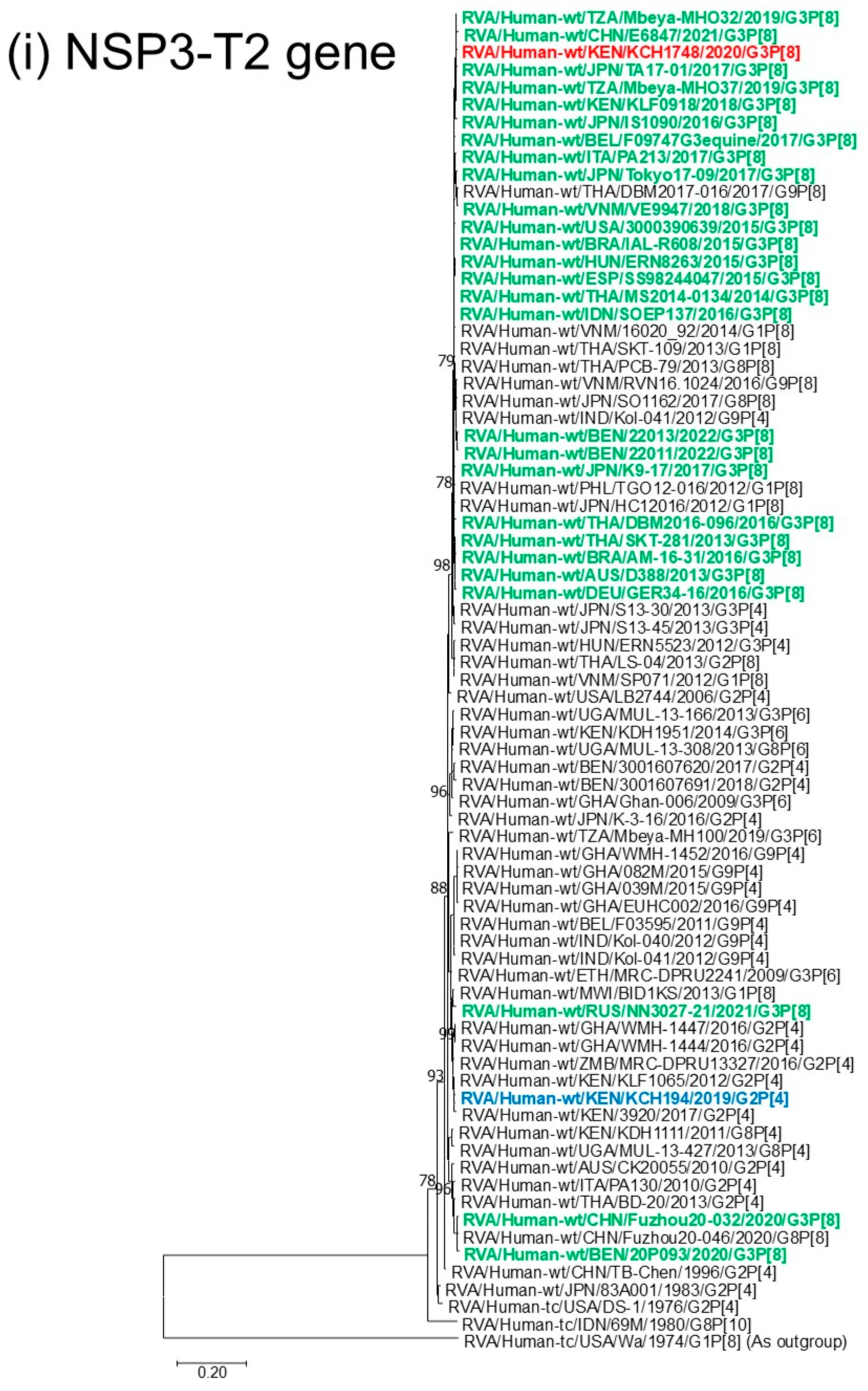

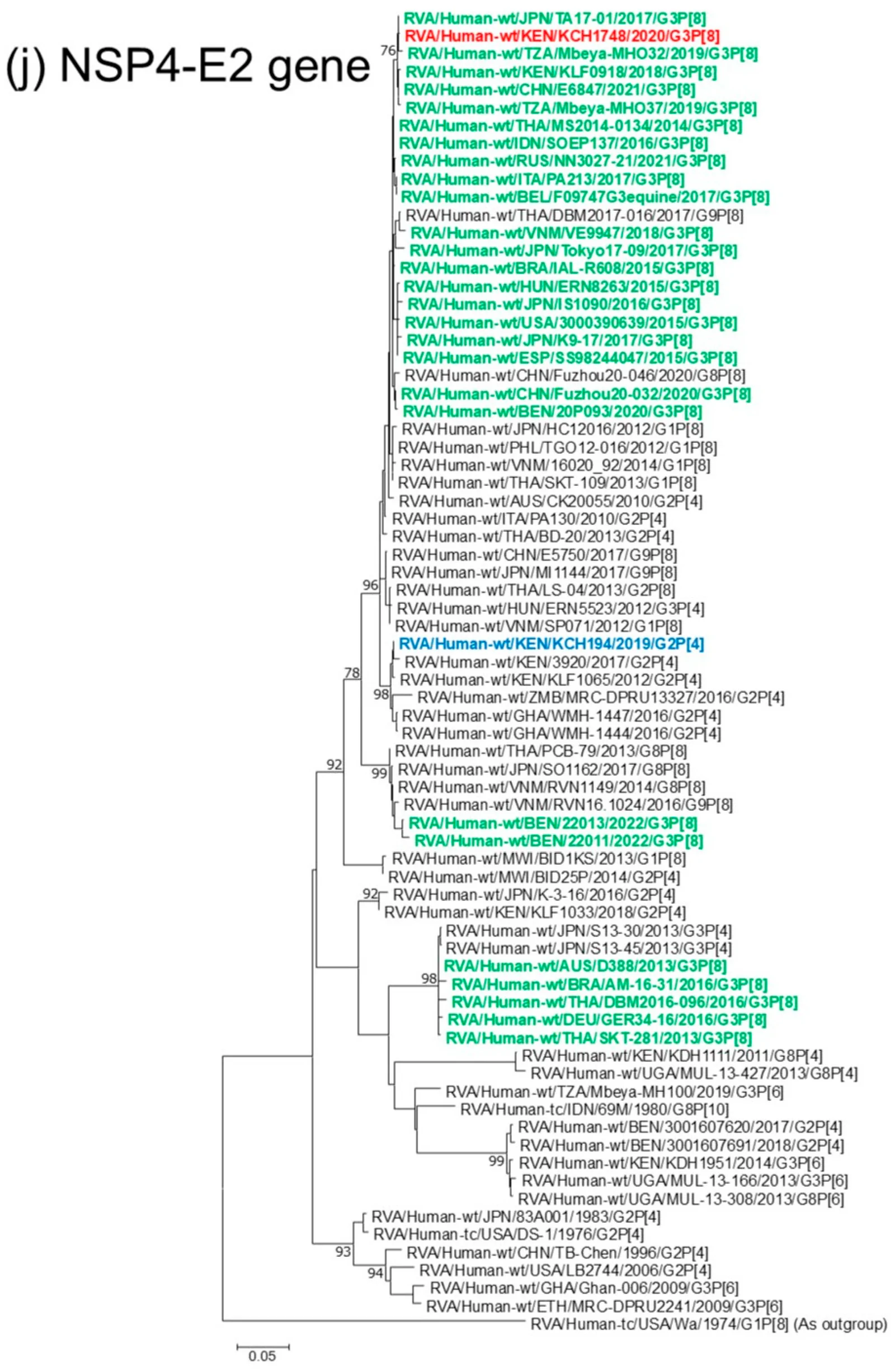

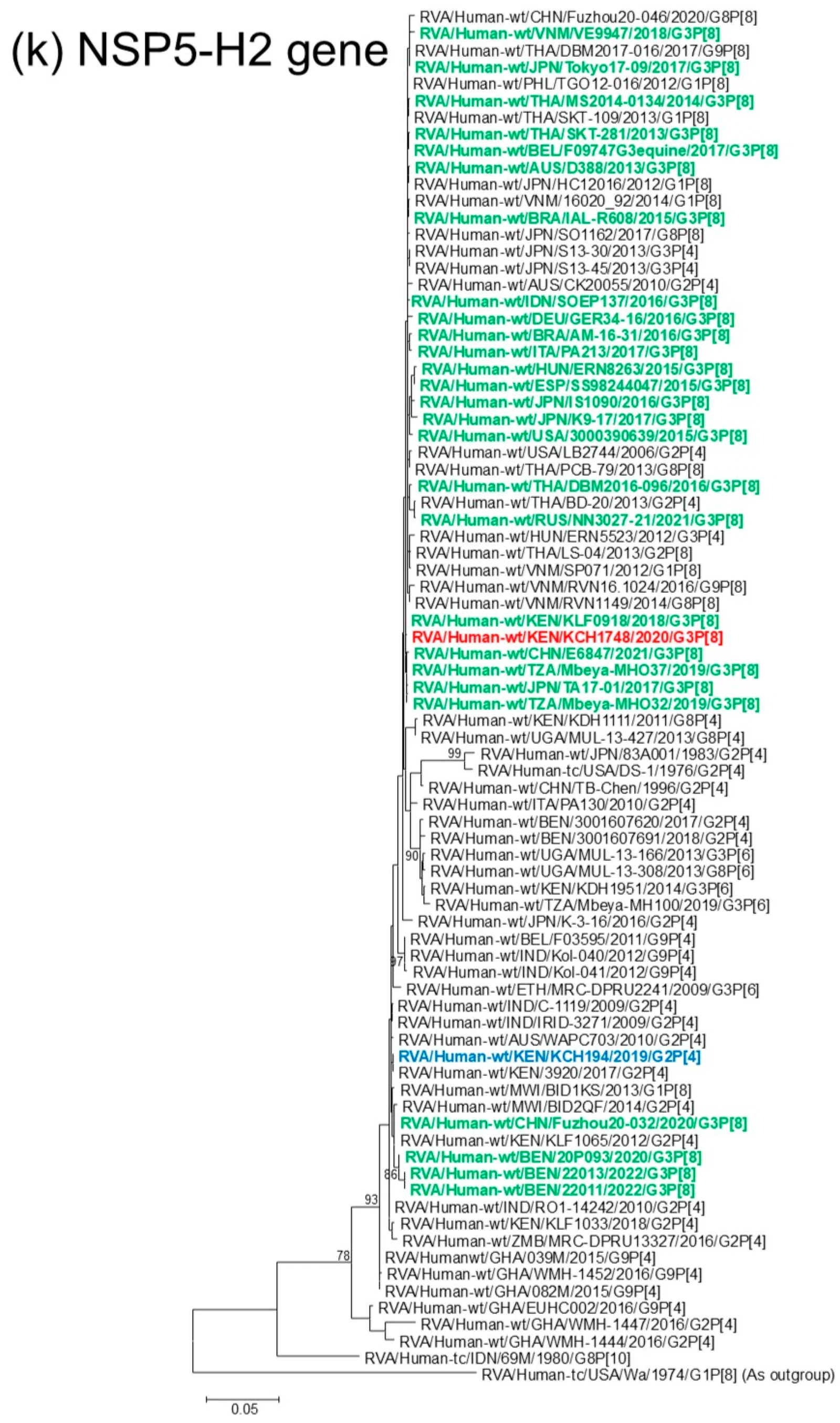
Phylogenetic trees constructed from the nucleotide sequences of the VP7-G3 (**a**), VP4-P[8] (**b**), VP6-I2 (**c**), VP1-R2 (**d**), VP2-C2 (**e**), VP3-M2 (**f**), NSP1-A2 (**g**), NSP2-N2 (**h**), NSP3-T2 (**i**), NSP4-E2 (**j**), and NSP5-H2 (**k**) genomic segments of the Kenyan DS-1-like G3P[8] strain KCH1748 and representative human RVA strains. The positions of KCH1748 are shown in red, whereas those of the other DS-1-like G3P[8] strains are shown in green. The positions of the co-circulating Wa-like G3P[8] strain KCH1884 and DS-1-like G2P[4] strain KCH194 are shown in blue. Bootstrap values of <75% are not shown. Scale bars indicate 0.02 (**b**), 0.05 (**h**,**j**,**k**), 0.10 (**a**,**c**,**e**,**g**), and 0.20 (**d**,**i**,**f**) substitutions per nucleotide.

To further characterize strain KCH1748, phylogenetic analyses were performed using the full-length nucleotide sequences of all 11 genomic segments (Figure 1). The reference dataset for these analyses was constructed by incorporating representative DS-1-like G3P[8] strains from prior whole-genome studies, closely related sequences identified through BLAST searches, and phylogenetically relevant African RVA strains. The nucleotide sequence similarities between strain KCH1748 and closely related RVA strains for each gene are shown in Table 2.

Eight of the 11 genomic segments (VP7, VP4, VP6, VP2, VP3, NSP2, NSP3, and NSP5) of strain KCH1748 shared the highest nucleotide sequence identities (99.7–100%) with those of East African DS-1-like G3P[8] strains, specifically the Kenyan strain KLF0918 and the Tanzanian strains Mbeya-MHO32 and Mbeya-MHO37 (Table 2) [31,32]. In contrast, the NSP1 and NSP4 genomic segments of strain KCH1748 shared the highest nucleotide sequence identities (99.7%) with the Japanese DS-1-like G3P[8] strain TA17-01 (Table 2). Phylogenetically, these 10 genomic segments were most closely related to the East African DS-1-like G3P[8] strains from Kenya and Tanzania, forming a common branch that also included the Japanese strain TA17-01 and the Chinese strain E6847, within the broader clade of globally circulating DS-1-like G3P[8] strains (Figure 1a–c,e–k).

In contrast to the other 10 genomic segments, the VP1 genomic segment of strain KCH1748 shared the highest nucleotide sequence identities (99.3%) with those of Ghanaian G9P[4] strains 039M and 082M (Table 2) [40]. Phylogenetically, the VP1 gene of strain KCH1748 was very closely related to these Ghanaian G9P[4] strains, sharing a well-supported common branch (bootstrap value = 100%) with Beninese DS-1-like G3P[8] strains 20P093 and 22011 and G2P[4] strains 3001607620 and 3001607691 (Figure 1d) [30].

Taken together, phylogenetic analysis showed that 10 of the 11 genomic segments of strain KCH1748 were closely related to East African DS-1-like G3P[8] strains from Kenya and Tanzania, whereas the VP1 gene clustered with Ghanaian G9P[4] strains.

Phylogenetic analysis further demonstrated that the VP7 and VP4 genomic segments of strain KCH1748 are genetically distinct from those of co-circulating Wa-like G3P[8] strain KCH1884. Similarly, its DS-1-like genomic backbone segments (I2-R2-C2-M2-A2-N2-T2-E2-H2) clustered distantly from those of the co-circulating DS-1-like G2P[4] strain KCH194. These findings indicate that KCH1748 is unlikely to have arisen from recent reassortment with these co-circulating strains.

**Figure 2:**
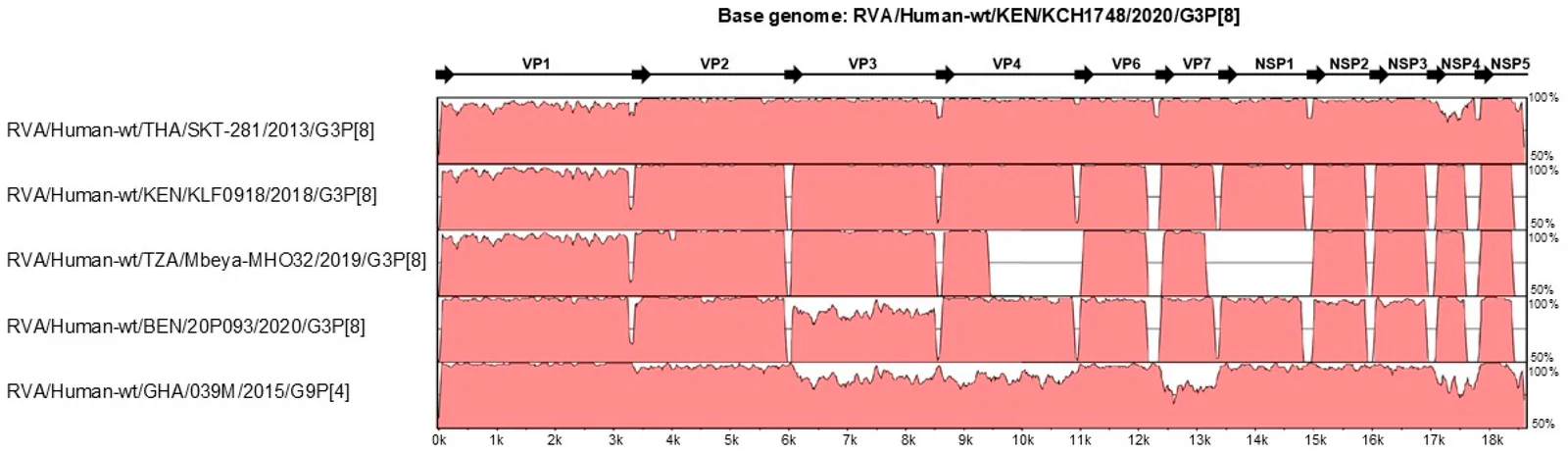
Nucleotide sequence similarities between the concatenated genome sequence of strain KCH1748 and those of representative RVA strains: the Thai prototype DS-1-like G3P[8] strain SKT-281, the Kenyan DS-1-like G3P[8] strain KLF0918, the Tanzanian DS-1-like G3P[8] strain Mbeya-MHO32, the Beninese DS-1-like G3P[8] strain 20P093, and the Ghanaian G9P[4] strain 039M. For strain Mbeya-MHO32, the VP4 sequence is partial and the NSP1 sequence is not available. Strain names are indicated on the left, and the positions of the 11 genomic segments are shown at the top. The bottom scale denotes the distance in kilobases (kb). The sequence-based percent similarity (50–100%) between strain KCH1748 and each reference strain is indicated on the vertical axis of each plot. Shaded regions indicate conserved sequences, and unshaded regions indicate regions with low sequence similarity or no corresponding sequence in reference strains.

To visually confirm the genomic composition of strain KCH1748, its concatenated genome sequence was compared with those of representative RVA strains using an mVISTA sequence similarity plot (Figure 2). This analysis showed that 10 out of 11 genomic segments of strain KCH1748 were highly conserved with those of representative DS-1-like G3P[8] strains such as the Thai prototype strain SKT-281, the Kenyan strain KLF0918, and the Tanzanian strain Mbeya-MHO32 [19,31,32]. In contrast, the VP1 genomic segment of KCH1748 shared higher genetic similarity with the Ghanaian G9P[4] strain 039M and the Beninese DS-1-like G3P[8] strain 20P093 [30,40]. Taken together, the mVISTA analysis supported the phylogenetic findings and showed that strain KCH1748 possesses a genomic composition largely consistent with those of the Thai, Kenyan, and Tanzanian DS-1-like G3P[8] strains, whereas its VP1 genomic segment shared higher similarity to the Ghanaian G9P[4] and Beninese DS-1-like G3P[8] strains.

## 4. Discussion

In the present study, we sequenced and analyzed the whole genome of the DS-1-like G3P[8] RVA strain KCH1748, which was detected in a child with AGE in Kenya. Although the complete genome sequence of an earlier Kenyan DS-1-like G3P[8] strain, KLF0918, is available in a public database, a detailed comparative phylogenetic characterization across all 11 genomic segments of this emerging lineage in Kenya has not previously been reported. To our knowledge, this study provides the first comprehensive whole-genome evolutionary characterization of a DS-1-like G3P[8] strain identified in Kenya by comparing KCH1748 with locally co-circulating strains and publicly available reference strains. Strain KCH1748 possessed an unusual genotype constellation, G3-P[8]-I2-R2-C2-M2-A2-N2-T2-E2-H2, comprising Wa-like outer capsid genes and a DS-1-like genomic backbone, consistent with DS-1-like G3P[8] strains circulating globally (Table 1). Phylogenetic analysis revealed that 10 of the 11 genomic segments of strain KCH1748 were closely related to those of other East African DS-1-like G3P[8] strains from Kenya and Tanzania, which clustered with the globally circulating DS-1-like G3P[8] lineage [31,32]. In contrast, the co-circulating Wa-like G3P[8] strain KCH1884 and DS-1-like G2P[4] strain KCH194 were phylogenetically distinct from strain KCH1748. Thus, these results suggest that strain KCH1748 belongs to the globally circulating DS-1-like G3P[8] lineage rather than having arisen through recent reassortment with these co-circulating strains in Kenya.

Notably, the VP1 gene of strain KCH1748 was most closely related to those of Ghanaian G9P[4] strains such as 039M and 082M [40], sharing a common branch with Beninese DS-1-like G3P[8] strains and G2P[4] strains [30]. These phylogenetic findings suggest that related VP1 lineages may be circulating among African RVA strains. However, strain KCH1748 was not closely related to the Beninese DS-1-like G3P[8] strains across the other genomic segments, indicating that strain KCH1748 is unlikely to be a direct descendant of Beninese DS-1-like G3P[8] strains. Rather, the VP1 gene of this strain may reflect an intragenogroup reassortment event involving G9P[4]-related strains circulating in Africa. Alternatively, strain KCH1748 may have inherited its VP1 gene through an intragenogroup reassortment event from an unobserved ancestral lineage shared with the Ghanaian and Beninese RVA strains. Although the precise geographic origin of this VP1 lineage cannot be determined because of the limited availability of African RVA sequences, and the identity of the donor strain and the location of the reassortment event also remain unknown, these findings indicate further genomic diversification among East African DS-1-like G3P[8] strains.

Although DS-1-like G3P[8] strains have recently emerged in Africa, their documented spread across the continent remains limited. To date, only eight DS-1-like G3P[8] strains have been identified in Africa, including strain KCH1748 described in this study, with detections in Kenya (2018), Tanzania (2019), and Benin (2020 and 2022) [30,31,32]. Notably, strain KCH1748 was the only strain with a short RNA electropherotype among the 80 available G3P[8] strains subjected to PAGE analysis, suggesting that DS-1-like G3P[8] strains constituted a minor subset of the G3P[8] strains examined in Central Kenya during the study period. These observations do not allow us to distinguish between sporadic introductions and low-level circulation that may have preceded widespread circulation of the lineage in the region. Therefore, prospective surveillance is needed to clarify the geographic distribution and transmission dynamics of DS-1-like G3P[8] strains in Africa.

Strain KCH1748 was detected in a child who had completed the two-dose Rotarix^®^ schedule. Similarly, Beninese DS-1-like G3P[8] strains were identified in children vaccinated with Rotavac^®^ (G9P[11]; Bharat Biotech, Telangana, India) [30]. While these findings indicate that DS-1-like G3P[8] infections can occur in vaccinated individuals, these data alone do not allow direct inference of vaccine evasion or reduced vaccine effectiveness; further studies are needed to verify these possibilities. Notably, the national immunization program in Kenya transitioned from Rotarix^®^ to Rotavac^®^ in 2023 [41]. This recent vaccine shift underscores the need for continued genomic surveillance to monitor circulating strains and assess the long-term impact of vaccination programs on RVA diversity in the region.

This study has several limitations. Because it is based on a single unusual DS-1-like G3P[8] strain, the findings should be interpreted cautiously and cannot be generalized to the broader circulation of this lineage in Kenya or elsewhere in Africa. In addition, the limited availability of whole-genome RVA sequences from Africa precluded identification of the direct donor strain and reconstruction of the precise reassortment history of the VP1 segment. Further prospective whole-genome surveillance across multiple locations will be required to better define the prevalence, transmission patterns, and genomic diversity of DS-1-like G3P[8] strains in Africa.

## 5. Conclusions

The whole-genome characterization of strain KCH1748 provides insights into the evolutionary dynamics of East African DS-1-like G3P[8] strains and suggests that intragenogroup reassortment may contribute to genomic diversification within this lineage. Continued genomic surveillance across multiple locations is needed to better assess the genomic diversity and transmission dynamics of DS-1-like G3P[8] strains across Africa.

## Figures and Tables

**Table 1:** Comparison of the genotype constellations of strain KCH1748 with those of representative human RVA strains.

Strain	VP7	VP4	VP6	VP1	VP2	VP3	NSP1	NSP2	NSP3	NSP4	NSP5
RVA/Human-wt/KEN/KCH1748/2020/G3P[8]	G3	P[8]	I2	R2	C2	M2	A2	N2	T2	E2	H2
RVA/Human-tc/USA/Wa/1974/G1P[8]	G1	P[8]	I1	R1	C1	M1	A1	N1	T1	E1	H1
RVA/Human-wt/JPN/HC12016/2012/G1P[8]	G1	P[8]	I2	R2	C2	M2	A2	N2	T2	E2	H2
RVA/Human-wt/PHL/TGO12-016/2012/G1P[8]	G1	P[8]	I2	R2	C2	M2	A2	N2	T2	E2	H2
RVA/Human-wt/VNM/SP071/2012/G1P[8]	G1	P[8]	I2	R2	C2	M2	A2	N2	T2	E2	H2
RVA/Human-wt/MWI/BID1KS/2013/G1P[8]	G1	P[8]	I2	R2	C2	M2	A2	N2	T2	E2	H2
RVA/Human-wt/THA/SKT-109/2013/G1P[8]	G1	P[8]	I2	R2	C2	M2	A2	N2	T2	E2	H2
RVA/Human-wt/VNM/16020_92/2014/G1P[8]	G1	P[8]	I2	R2	C2	M2	A2	N2	T2	E2	H2
RVA/Human-tc/USA/DS-1/1976/G2P[4]	G2	P[4]	I2	R2	C2	M2	A2	N2	T2	E2	H2
RVA/Human-wt/CHN/TB-Chen/1996/G2P[4]	G2	P[4]	I2	R2	C2	M2	A2	N2	T2	E2	H2
RVA/Human-wt/AUS/CK20030/2006/G2P[4]	G2	P[4]	I2	R2	C2	M2	A2	N2	T2	E2	H2
RVA/Human-wt/USA/LB2744/2006/G2P[4]	G2	P[4]	I2	R2	C2	M2	A2	N2	T2	E2	H2
RVA/Human-wt/MWI/BID25P/2014/G2P[4]	G2	P[4]	I2	R2	C2	M2	A2	N2	T2	E2	H2
RVA/Human-wt/ZMB/MRC-DPRU13327/2016/G2P[4]	G2	P[4]	I2	R2	C2	M2	A2	N2	T2	E2	H2
RVA/Human-wt/GHA/WMH-1444/2016/G2P[4]	G2	P[4]	I2	R2	C2	M2	A2	N2	T2	E2	H2
RVA/Human-wt/BEN/3001607620/2017/G2P[4]	G2	P[4]	I2	R2	C2	M2	A2	N2	T2	E2	H2
RVA/Human-wt/KEN/3920/2017/G2P[4]	G2	P[4]	I2	R2	C2	M2	A2	N2	T2	E2	H2
RVA/Human-wt/BEN/3001607691/2018/G2P[4]	G2	P[4]	I2	R2	C2	M2	A2	N2	T2	E2	H2
RVA/Human-wt/KEN/KCH194/2019/G2P[4]	G2	P[4]	I2	R2	C2	M2	A2	N2	T2	E2	H2
RVA/Human-wt/JPN/S13-30/2013/G3P[4]	G3	P[4]	I2	R2	C2	M2	A2	N2	T2	E2	H2
RVA/Human-wt/GHA/Ghan-006/2009/G3P[6]	G3	P[6]	I2	R2	C2	M2	A2	N2	T2	E2	H2
RVA/Human-wt/ETH/MRC-DPRU2241/2009/G3P[6]	G3	P[6]	I2	R2	C2	M2	A2	N2	T2	E2	H2
RVA/Human-wt/UGA/MUL-13-166/2013/G3P[6]	G3	P[6]	I2	R2	C2	M2	A2	N2	T2	E2	H2
RVA/Human-wt/KEN/KDH1951/2014/G3P[6]	G3	P[6]	I2	R2	C2	M2	A2	N2	T2	E2	H2
RVA/Human-wt/TZA/Mbeya-MH100/2019/G3P[6]	G3	P[6]	I2	R2	C2	M2	-	N2	T2	E2	H2
RVA/Human-tc/USA/P/1974/G3P[8]	G3	P[8]	I1	R1	C1	M1	A1	N1	T1	E1	H1
RVA/Human-tc/USA/DC23/1976/G3P[8]	G3	P[8]	I1	R1	C1	M1	A1	N1	T1	E1	H1
RVA/Human-tc/JPN/YO/1976/G3P[8]	G3	P[8]	I1	R1	C1	M1	A1	N1	T1	E1	H1
RVA/Human-wt/CHN/R1604/2011/G3P[8]	G3	P[8]	I1	R1	C1	M1	A1	N1	T1	E1	H1
RVA/Human-wt/MWI/BTY2BD/2018/G3P[8]	G3	P[8]	I1	R1	C1	M1	A1	N1	T1	E1	H1
RVA/Human-wt/KEN/KLF1002/2018/G3P[8]	G3	P[8]	I1	R1	C1	M1	A1	N1	T1	E1	H1
RVA/Human-wt/KEN/KCH1884/2020/G3P[8]	G3	P[8]	I1	R1	C1	M1	A1	N1	T1	E1	H1
RVA/Human-wt/MOZ/MAN1811450.8/2021/G3P[8]	G3	P[8]	I1	R1	C1	M1	A1	N1	T1	E1	H1
RVA/Human-wt/AUS/D388/2013/G3P[8]	G3	P[8]	I2	R2	C2	M2	A2	N2	T2	E2	H2
RVA/Human-wt/THA/SKT-281/2013/G3P[8]	G3	P[8]	I2	R2	C2	M2	A2	N2	T2	E2	H2
RVA/Human-wt/THA/MS2014-0134/2014/G3P[8]	G3	P[8]	I2	R2	C2	M2	A2	N2	T2	E2	H2
RVA/Human-wt/USA/3000390639/2015/G3P[8]	G3	P[8]	I2	R2	C2	M2	A2	N2	T2	E2	H2
RVA/Human-wt/HUN/ERN8263/2015/G3P[8]	G3	P[8]	I2	R2	C2	M2	A2	N2	T2	E2	H2
RVA/Human-wt/BRA/IAL-R608/2015/G3P[8]	G3	P[8]	I2	R2	C2	M2	A2	N2	T2	E2	H2
RVA/Human-wt/ESP/SS98244047/2015/G3P[8]	G3	P[8]	I2	R2	C2	M2	A2	N2	T2	E2	H2
RVA/Human-wt/BRA/AM-16-31/2016/G3P[8]	G3	P[8]	I2	R2	C2	M2	A2	N2	T2	E2	H2
RVA/Human-wt/THA/DBM2016-096/2016/G3P[8]	G3	P[8]	I2	R2	C2	M2	A2	N2	T2	E2	H2
RVA/Human-wt/DEU/GER34-16/2016/G3P[8]	G3	P[8]	I2	R2	C2	M2	A2	N2	T2	E2	H2
RVA/Human-wt/JPN/IS1090/2016/G3P[8]	G3	P[8]	I2	R2	C2	M2	A2	N2	T2	E2	H2
RVA/Human-wt/IDN/SOEP137/2016/G3P[8]	G3	P[8]	I2	R2	C2	M2	A2	N2	T2	E2	H2
RVA/Human-wt/BEL/F09747G3equine/2017/G3P[8]	G3	P[8]	I2	R2	C2	M2	A2	N2	T2	E2	H2
RVA/Human-wt/JPN/K9-17/2017/G3P[8]	G3	P[8]	I2	R2	C2	M2	A2	N2	T2	E2	H2
RVA/Human-wt/ITA/PA213/2017/G3P[8]	G3	P[8]	I2	R2	C2	M2	A2	N2	T2	E2	H2
RVA/Human-wt/JPN/TA17-01/2017/G3P[8]	G3	P[8]	I2	R2	C2	M2	A2	N2	T2	E2	H2
RVA/Human-wt/JPN/Tokyo17-09/2017/G3P[8]	G3	P[8]	I2	R2	C2	M2	A2	N2	T2	E2	H2
RVA/Human-wt/KEN/KLF0918/2018/G3P[8]	G3	P[8]	I2	R2	C2	M2	A2	N2	T2	E2	H2
RVA/Human-wt/VNM/VE9947/2018/G3P[8]	G3	P[8]	I2	R2	C2	M2	A2	N2	T2	E2	H2
RVA/Human-wt/TZA/Mbeya-MHO32/2019/G3P[8]	G3	P[8]	I2	R2	C2	M2	-	N2	T2	E2	H2
RVA/Human-wt/TZA/Mbeya-MHO37/2019/G3P[8]	G3	P[8]	I2	R2	C2	M2	-	N2	T2	E2	H2
RVA/Human-wt/BEN/20P093/2020/G3P[8]	G3	P[8]	I2	R2	C2	M2	A2	N2	T2	E2	H2
RVA/Human-wt/CHN/Fuzhou20-032/2020/G3P[8]	G3	P[8]	I2	R2	C2	M2	A2	N2	T2	E2	H2
RVA/Human-wt/CHN/E6847/2021/G3P[8]	G3	P[8]	I2	R2	C2	M2	A2	N2	T2	E2	H2
RVA/Human-wt/RUS/NN3027-21/2021/G3P[8]	G3	P[8]	I2	R2	C2	M2	A2	N2	T2	E2	H2
RVA/Human-wt/BEN/22011/2022/G3P[8]	G3	P[8]	I2	R2	C2	M2	A2	N2	T2	E2	H2
RVA/Human-wt/BEN/22013/2022/G3P[8]	G3	P[8]	I2	R2	C2	M2	A2	N2	T2	E2	H2
RVA/Human-wt/VEN/3-CLC/2023/G3P[8]	G3	P[8]	I2	R2	C2	M2	A2	N2	T2	E2	H2
RVA/Human-wt/KEN/KDH1111/2011/G8P[4]	G8	P[4]	I2	R2	C2	M2	A2	N2	T2	E2	H2
RVA/Human-wt/UGA/MUL-13-427/2013/G8P[4]	G8	P[4]	I2	R2	C2	M2	A2	N2	T2	E2	H2
RVA/Human-wt/UGA/MUL-13-308/2013/G8P[6]	G8	P[6]	I2	R2	C2	M2	A2	N2	T2	E2	H2
RVA/Human-wt/THA/NP-130/2014/G8P[8]	G8	P[8]	I2	R2	C2	M2	A2	N2	T2	E2	H2
RVA/Human-wt/VNM/RVN1149/2014/G8P[8]	G8	P[8]	I2	R2	C2	M2	A2	N2	T2	E2	H2
RVA/Human-wt/JPN/To14-0/2014/G8P[8]	G8	P[8]	I2	R2	C2	M2	A2	N2	T2	E2	H2
RVA/Human-wt/CHN/Fuzhou20-046/2020/G8P[8]	G8	P[8]	I2	R2	C2	M2	A2	N2	T2	E2	H2
RVA/Human-wt/IND/Kol-040/2012/G9P[4]	G9	P[4]	I2	R2	C2	M2	A2	N2	T2	E6	H2
RVA/Human-wt/IND/Kol-041/2012/G9P[4]	G9	P[4]	I2	R2	C2	M2	A2	N2	T2	E6	H2
RVA/Human-wt/GHA/039M/2015/G9P[4]	G9	P[4]	I2	R2	C2	M2	A2	N2	T2	E6	H2
RVA/Human-wt/GHA/082M/2015/G9P[4]	G9	P[4]	I2	R2	C2	M2	A2	N2	T2	E6	H2
RVA/Human-wt/GHA/EUHC002/2016/G9P[4]	G9	P[4]	I2	R2	C2	M2	A2	N2	T2	E6	H2
RVA/Human-wt/GHA/WMH-1452/2016/G9P[4]	G9	P[4]	I2	R2	C2	M2	A2	N2	T2	E6	H2
RVA/Human-wt/VNM/RVN16.1024/2016/G9P[8]	G9	P[8]	I2	R2	C2	M2	A2	N2	T2	E2	H2
RVA/Human-wt/THA/DBM2017-203/2017/G9P[8]	G9	P[8]	I2	R2	C2	M2	A2	N2	T2	E2	H2

The Kenyan DS-1-like G3P[8] strain (KCH1748) is shown in red. The co-circulating reference strains (KCH1884 and KCH194) are shown in blue. Representative DS-1-like G3P[8] strains identified previously are shown in green. Gray shading indicates the genomic segments with genotypes identical to those of the Kenyan DS-1-like G3P[8] strain KCH1748. A dash (-) indicates an undetermined genotype.

**Table 2:** Nucleotide sequence identities between Kenyan DS-1-like G3P[8] strain KCH1748 and closely related RVA strains for each genomic segment.

Gene	Closely Related Strain(s)	Identity (%)	Accession Numbers	Reference
VP7	RVA/Human-wt/KEN/KLF0918/2018/G3P[8]	99.7	MZ096469	[32]
VP4	RVA/Human-wt/KEN/KLF0918/2018/G3P[8]	99.8	MZ096467	[32]
VP6	RVA/Human-wt/TZA/Mbeya-MHO32/2019/G3P[8] RVA/Human-wt/TZA/Mbeya-MHO37/2019/G3P[8]	99.7 99.7	ON058277 ON058278	[31] [31]
VP1	RVA/Human-wt/GHA/039M/2015/G9P[4] RVA/Human-wt/GHA/082M/2015/G9P[4]	99.3 99.3	OR890151 OR890152	[40] [40]
VP2	RVA/Human-wt/KEN/KLF0918/2018/G3P[8]	99.7	MZ096465	−
VP3	RVA/Human-wt/KEN/KLF0918/2018/G3P[8]	99.7	MZ096466	−
NSP1	RVA/Human-wt/JPN/TA17-01/2017/G3P[8]	99.7	LC542509	−
NSP2	RVA/Human-wt/TZA/Mbeya-MHO32/2019/G3P[8] RVA/Human-wt/TZA/Mbeya-MHO37/2019/G3P[8]	99.8 99.8	ON092391 ON092392	[31] [31]
NSP3	RVA/Human-wt/KEN/KLF0918/2018/G3P[8] RVA/Human-wt/TZA/Mbeya-MHO32/2019/G3P[8] RVA/Human-wt/TZA/Mbeya-MHO37/2019/G3P[8]	99.9 99.9 99.9	MZ096461 ON092381 ON092382	− [31] [31]
NSP4	RVA/Human-wt/JPN/TA17-01/2017/G3P[8]	99.7	LC542512	−
NSP5	RVA/Human-wt/KEN/KLF0918/2018/G3P[8] RVA/Human-wt/TZA/Mbeya-MHO32/2019/G3P[8]	100.0 100.0	MZ096463 ON109522	− [31]

−, no associated publications are available.

## Data Availability

The nucleotide sequence data obtained in this study have been deposited in the DDBJ/ENA/GenBank databases. The accession numbers for the nucleotide sequences of the VP1–VP4, VP6, VP7, and NSP1–NSP5 genomic segments of the study strains are LC940160–LC940170 for KCH1748, LC940171–LC940181 for KCH1884, and LC940182–LC940192 for KCH194, respectively.

## Supplementary Materials

The following supporting information can be downloaded at https://www.mdpi.com/article/10.3390/v18090930/s1, Table S1: Sequencing data for the 11 genomic segments of the Kenyan study strains (KCH1748, KCH1884, and KCH194).
