# Supplementary material for: The First Whole-Genome Characterization of a Kenyan DS-1-like G3P[8] Rotavirus Strain: Evidence for an Intragenogroup Reassortment Event in Africa
Source: Viruses. 2026 Aug 24;18(9):930. doi: 10.3390/v18090930 (PMC13611889; doi:10.3390/v18090930)
Supplement: Supplementary file 1 [file viruses-18-00930-s001.zip › viruses-4460462-supplementary.pdf]

Supplementary Table S1

Sequencing data for the 11 genomic segments of the Kenyan study strains (KCH1748, KCH1884, and KCH194).

Study strain	Total reads[a]		Gene										
			VP7	VP4	VP6	VP1	VP2	VP3	NSP1	NSP2	NSP3	NSP4	NSP5
RVA/Human-wt/KEN/KCH1748/2020/G3P[8]	1,534,622	Nucleotides; bp (% coverage of the full-length)	1,062 (100%)	2,359 (100%)	1,356 (100%)	3,302 (100%)	2,687 (100%)	2,591 (100%)	1,566 (100%)	1,059 (100%)	1,066 (100%)	751 (100%)	816 (100%)
		Deduced amino acids; aa (% coverage of the full-length)	326 (100%)	775 (100%)	397 (100%)	1,088 (100%)	879 (100%)	835 (100%)	486 (100%)	317 (100%)	313 (100%)	175 (100%)	200 (100%)
		Reads mapped to gene segment	59,234	881	15,768	157,236	11,529	59,270	177,474	84,114	522,562	27,910	116,843
		Maximum depth of reads	3,587	460	741	2,954	503	1,402	7,511	4,519	28,335	2,336	9,093
RVA/Human-wt/KEN/KCH1884/2020/G3P[8]	899,754	Nucleotides; bp (% coverage of the full-length)	1,062 (100%)	2,359 (100%)	1,356 (100%)	3,292 (99.7%)	2,729 (100%)	2,586 (99.8%)	1,563 (99.7%)	1,049 (99.1%)	1,069 (99.5%)	743 (98.9%)	601 (90.5%)
		Deduced amino acids; aa (% coverage of the full-length)	326 (100%)	775 (100%)	397 (100%)	1,088 (100%)	894 (100%)	835 (100%)	486 (100%)	317 (100%)	310 (100%)	175 (100%)	197 (100%)
		Reads mapped to gene segment	13,042	26,649	8,591	25,959	28,129	23,597	17,783	10,371	16,795	12,613	1,202
		Maximum depth of reads	1,895	2,173	1,844	1,194	1,832	1,332	1,987	1,467	1,765	3,744	340
RVA/Human-wt/KEN/KCH194/2019/G2P[4]	1,921,126	Nucleotides; bp (% coverage of the full-length)	1,026 (96.6%)	2,352 (99.7%)	1,349 (99.5%)	3,294 (99.8%)	2,676 (99.6%)	2,586 (99.8%)	1,566 (100%)	1,050 (99.2%)	1,066 (100%)	735 (97.9%)	798 (97.8%)
		Deduced amino acids; aa (% coverage of the full-length)	326 (100%)	775 (100%)	397 (100%)	1,088 (100%)	879 (100%)	835 (100%)	486 (100%)	317 (100%)	313 (100%)	175 (100%)	200 (100%)
		Reads mapped to gene segment	21,235	38,831	16,213	54,537	44,720	37,112	15,070	23,340	21,356	7,059	5,144
		Maximum depth of reads	2,821	2,904	2,064	2,398	3,011	2,054	2,440	2,871	2,547	1,922	1,038

[a]Sequence reads remaining after adapter trimming.
